# Supplementary material for: Afrotropical montane birds experience upslope shifts and range contractions along a fragmented elevational gradient in response to global warming
Source: PLoS One. 2021 Mar 30;16(3):e0248712. doi: 10.1371/journal.pone.0248712 (PMC8009416; doi:10.1371/journal.pone.0248712)
Supplement: S1 Table — Number of captures are across both historic and current surveys. (DOCX) [file pone.0248712.s001.docx]

| **S1 Table.** | | | | | | | | | | | | | | | |
| --- | --- | --- | --- | --- | --- | --- | --- | --- | --- | --- | --- | --- | --- | --- | --- |
| **Species** | **Common name** | **Family** | **Number of captures** | **Historic elevational range (m)** | | | **Current elevational range (m)** | | | **Observed elevational range shift (m)** | | | **Corrected elevational range shift (m)** | | |
|  |  |  |  | Lower range limit | Mean | Upper range limit | Lower range limit | Mean | Upper range limit | Lower range limit | Mean | Upper range limit | Lower range limit | Mean | Upper range limit |
| *Aplopelia larvata* | Lemon Dove | Columbidae | 5 | 1545 | 1820 | 2096 | 1316 | 1420 | 1525 | -229 | -400 | -571 | -295 | -372 | -413 |
| *Apaloderma vittatum* | Bar-tailed Trogon | Trogonidae | 6 | 1310 | 1638 |  | 1827 | 1965 |  | 517 | 328 |  | 423 | 354 |  |
| *Batis mixta* | Forest Batis | Platysteiridae | 28 | 360 | 1197 | 1820 | 580 | 1259 | 2045 | 220 | 62 | 225 | 132 | 64 | 310 |
| *Dicrurus ludwigii* | Square-tailed Drongo | Dicruridae | 12 |  | 860 | 1503 |  | 628 | 1219 |  | -232 | -284 |  | -278 | -84 |
| *Elminia albonotata* | White-tailed Crested-flycatcher | Stenostiridae | 48 | 1259 | 1619 |  | 1310 | 1690 |  | 51 | 71 |  | 68 | 75 |  |
| *Artisornis metopias* | Red-capped Forest-warbler | Cisticolidae | 17 | 1321 | 1553 | 1806 | 1624 | 2027 | 2110 | 303 | 474 | 305 | 161 | 460 | 307 |
| *Apalis thoracica* | Bar-throated Apalis | Cisticolidae | 6 | 1348 | 1765 |  | 1545 | 1820 |  | 196 | 55 |  | 76 | 66 |  |
| *Arizelocichla striifacies* | Olive-headed Bulbul | Pycnonotidae | 38 | 1020 | 1351 | 1820 | 1020 | 1464 | 1820 | 0 | 113 | 0 | -9 | 142 | 46 |
| *Arizelocichla nigriceps* | Eastern Mountain Greenbul | Pycnonotidae | 28 | 1820 | 2038 |  | 2110 | 2110 |  | 290 | 73 |  | 252 | 65 |  |
| *Arizelocichla masukuensis* | Shelley's Greenbul | Pycnonotidae | 125 | 580 | 1405 |  | 1020 | 1497 |  | 440 | 92 |  | 369 | 106 |  |
| *Eurillas virens* | Little Greenbul | Pycnonotidae | 49 |  | 629 | 1020 |  | 912 | 1310 |  | 283 | 290 |  | 238 | 309 |
| *Phyllastrephus albigula* | Montane Tiny Greenbul | Pycnonotidae | 106 | 360 | 1497 |  | 580 | 1421 |  | 220 | -75 |  | 130 | -61 |  |
| *Phyllastrephus fischeri* | Fischer's Greenbul | Pycnonotidae | 4 |  | 360 | 360 |  | 360 | 360 |  | 0 | 0 |  | 0 | 0 |
| *Phyllastrephus cabanisi* | Cabanis's Greenbul | Pycnonotidae | 80 | 1020 | 1480 |  | 1020 | 1510 |  | 0 | 30 |  | -3 | 53 |  |
| *Phyllastrephus flavostriatus* | Yellow-streaked Greenbul | Pycnonotidae | 31 | 498 | 1089 | 1530 | 1020 | 1078 | 1281 | 523 | -11 | -249 | 422 | -26 | -181 |
| *Phylloscopus ruficapilla* | Yellow-throated Woodland-warbler | Phylloscopidae | 43 | 1180 | 1554 |  | 1158 | 1642 |  | -22 | 88 |  | -54 | 115 |  |
| *Illadopsis distans* | Grey-breasted Illadopsis | Pellorneidae | 18 |  | 916 | 1310 |  | 1091 | 1310 |  | 175 | 0 |  | 112 | 3 |
| *Geokichla gurneyi* | Orange Ground-thrush | Turdidae | 6 | 580 | 890 | 1727 | 1323 | 1565 | 1807 | 743 | 675 | 80 | 504 | 621 | 312 |
| *Turdus roehli* | Usambara Thrush | Turdidae | 48 | 1020 | 1822 |  | 1020 | 1524 |  | 0 | -298 |  | -24 | -281 |  |
| *Pogonocichla stellata* | White-starred Robin | Muscicapidae | 35 | 360 | 1386 |  | 1530 | 1874 |  | 1170 | 488 |  | 896 | 497 |  |
| *Chamaetylas fuelleborni* | White-chested Alethe | Muscicapidae | 54 | 580 | 1468 |  | 701 | 1335 |  | 121 | -133 |  | 31 | -131 |  |
| *Sheppardia montana* | Usambara Akalat | Muscicapidae | 46 | 1820 | 2005 |  | 1820 | 1932 |  | 0 | -73 |  | 0 | -81 |  |
| *Sheppardia sharpei* | Sharpe's Akalat | Muscicapidae | 65 | 580 | 1258 | 1530 | 932 | 1291 | 1530 | 352 | 33 | 0 | 219 | 42 | 0 |
| *Modulatrix stictigula* | Spot-throat | Modulatricidae | 56 | 1310 | 1693 |  | 1376 | 1758 |  | 66 | 65 |  | 45 | 61 |  |
| *Cyanomitra olivacea* | Olive Sunbird | Nectariniidae | 94 | 580 | 1268 | 1820 | 580 | 1248 | 1530 | 0 | -20 | -290 | -2 | -19 | -258 |
| *Hypargos niveoguttatus* | Red-throated Twinspot | Estrildidae | 6 |  | 360 | 360 |  | 360 | 360 |  | 0 | 0 |  | 0 | 0 |
| *Spermophaga ruficapilla* | Red-headed Bluebill | Estrildidae | 8 | 360 | 690 | 1020 | 1020 | 1020 | 1020 | 660 | 330 | 0 | 477 | 281 | 42 |
| *Cryptospiza reichenovii* | Red-faced Crimsonwing | Estrildidae | 21 | 1310 | 1557 | 1820 | 1310 | 1601 | 2110 | 0 | 44 | 290 | -5 | 76 | 375 |
| *Linurgus olivaceus* | Oriole Finch | Fringillidae | 18 | 1530 | 1584 | 1820 | 2110 | 2110 | 2110 | 580 | 526 | 290 | 576 | 502 | 381 |
