# Supplementary figures and images for: Afrotropical montane birds experience upslope shifts and range contractions along a fragmented elevational gradient in response to global warming
Source: PLoS One. 2021 Mar 30;16(3):e0248712. doi: 10.1371/journal.pone.0248712 (PMC8009416; doi:10.1371/journal.pone.0248712)

S1 Figure.

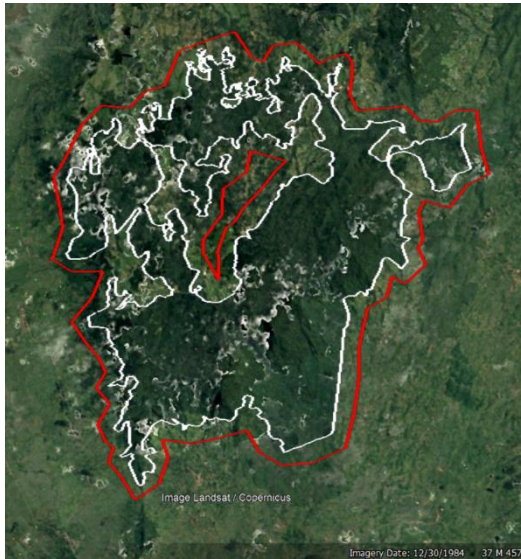

30 Dec 1984

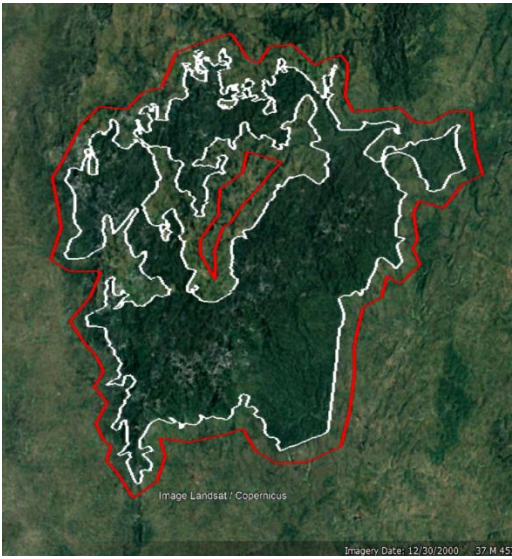

30 Dec 2000

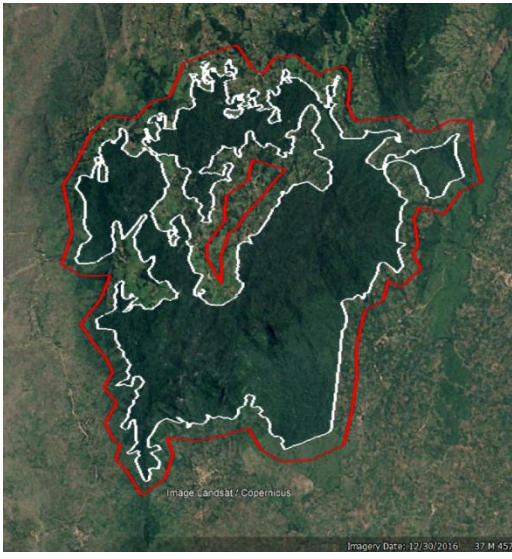

30 Dec 2016

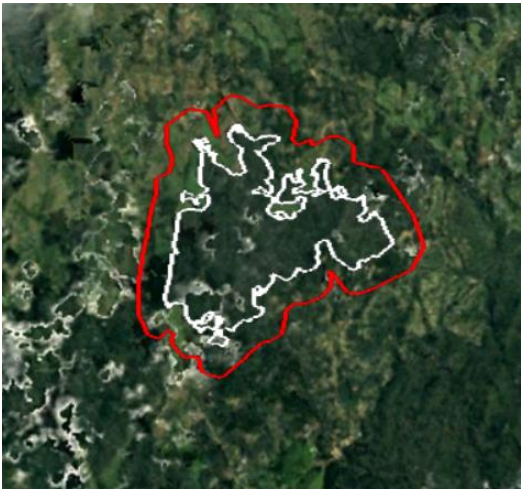

30 Dec 1984

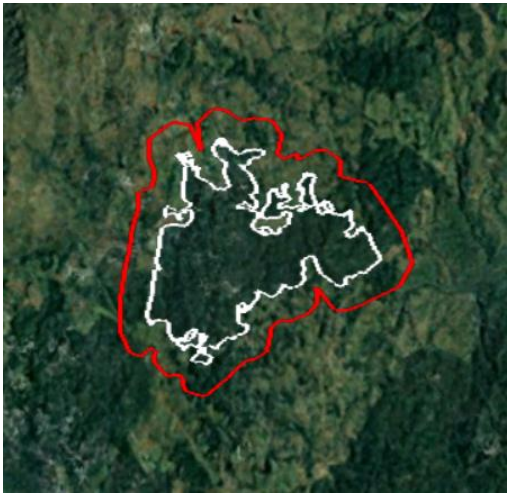

30 Dec 2000

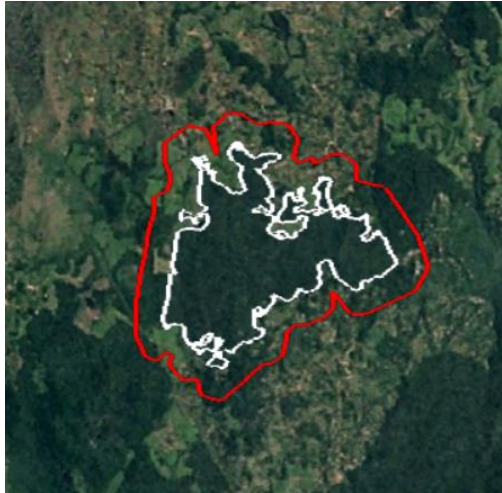

30 Dec 2016

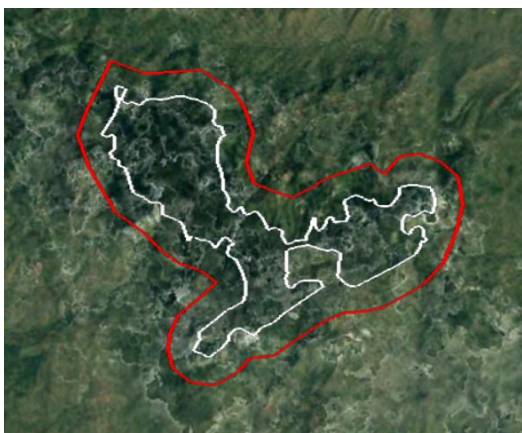

30 Dec 1985

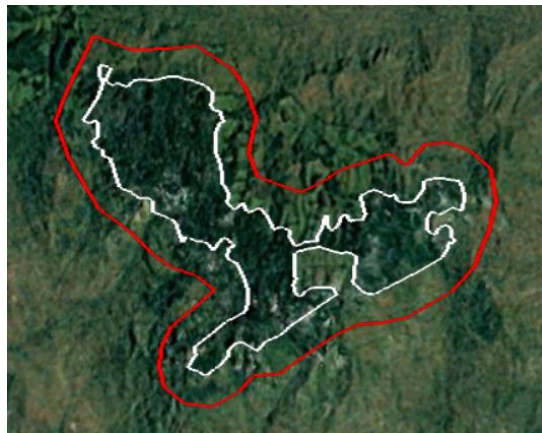

30 Dec 2001

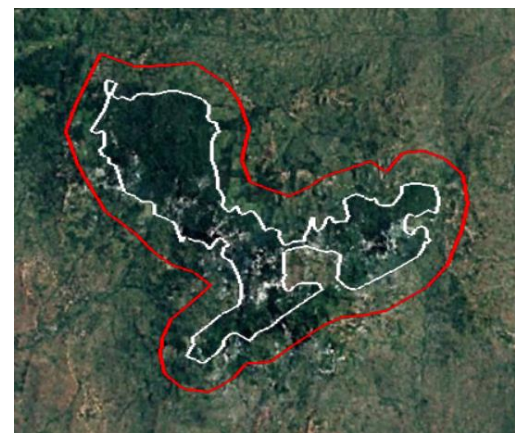

30 Dec 2016

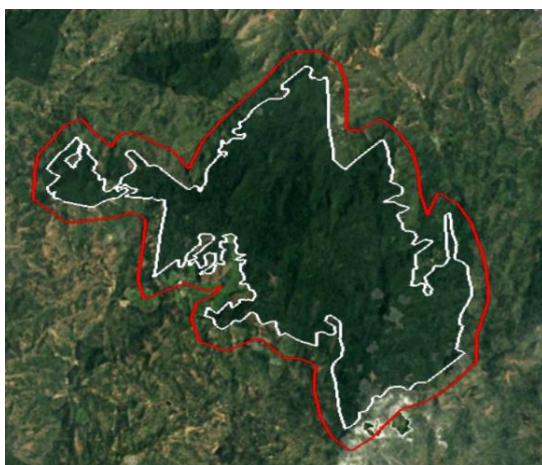

30 Dec 1987

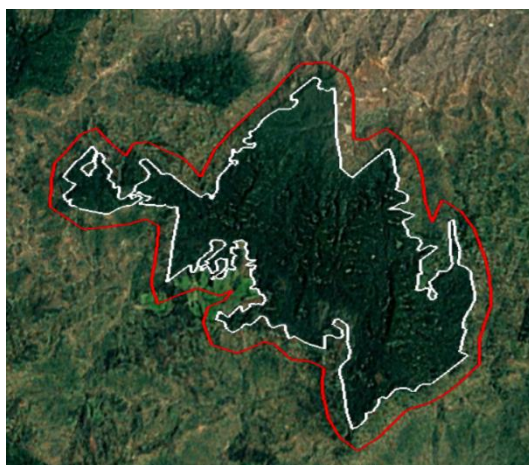

30 Dec 1999

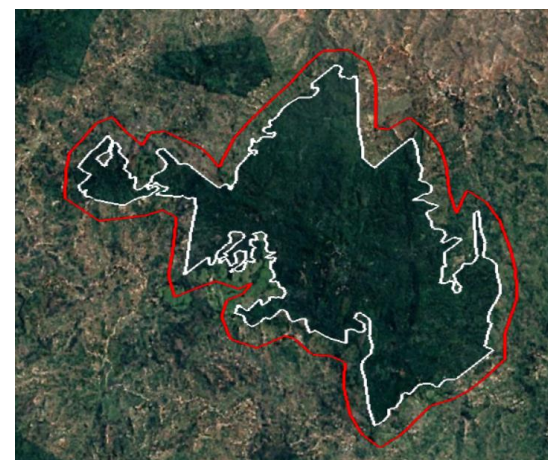

30 Dec 2016

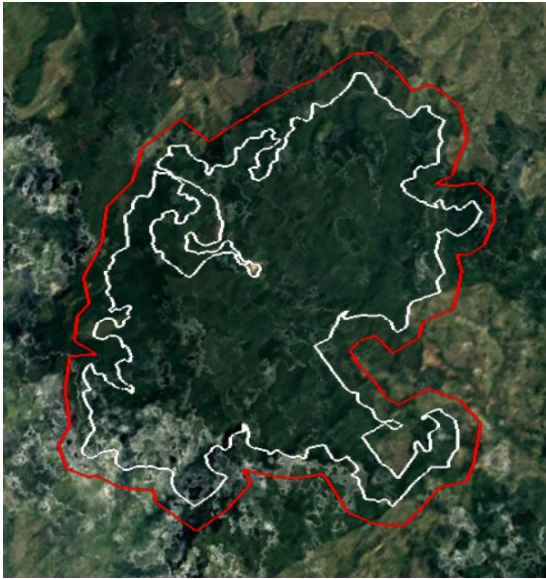

30 Dec 1985

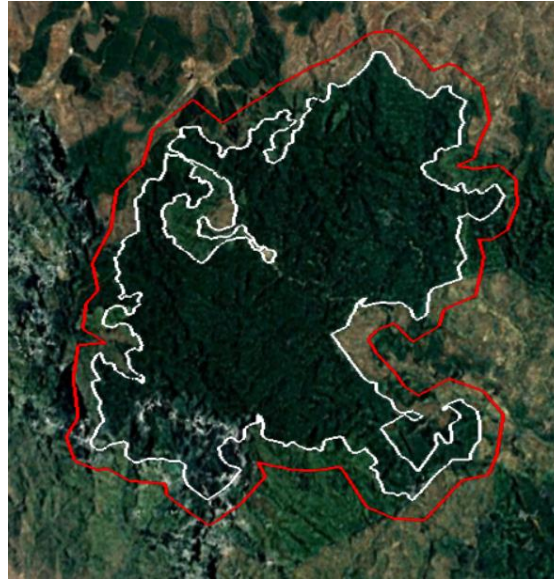

30 Dec 2000

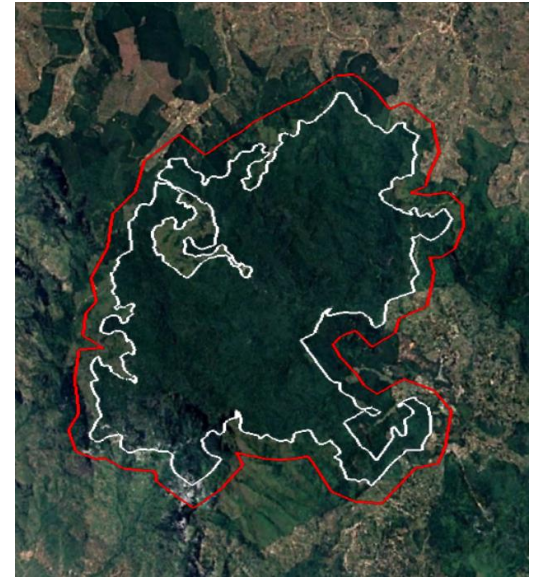

30 Dec 2016

Supplement: S1 Fig — The forest block displayed in row 1 contains survey sites at 360 m and 580 m; row 2 contains the survey site at 1020 m; row 3 contains the survey site at 1310 m; row 4 contains the survey site at 1530 m; and row 5 contains survey sites at 1820 m and 2110 m. The perimeter of each forest block is outlined in white, and matrix habitats within 500 m of the forest edge are outlined in red. (PDF) [file pone.0248712.s002.pdf]

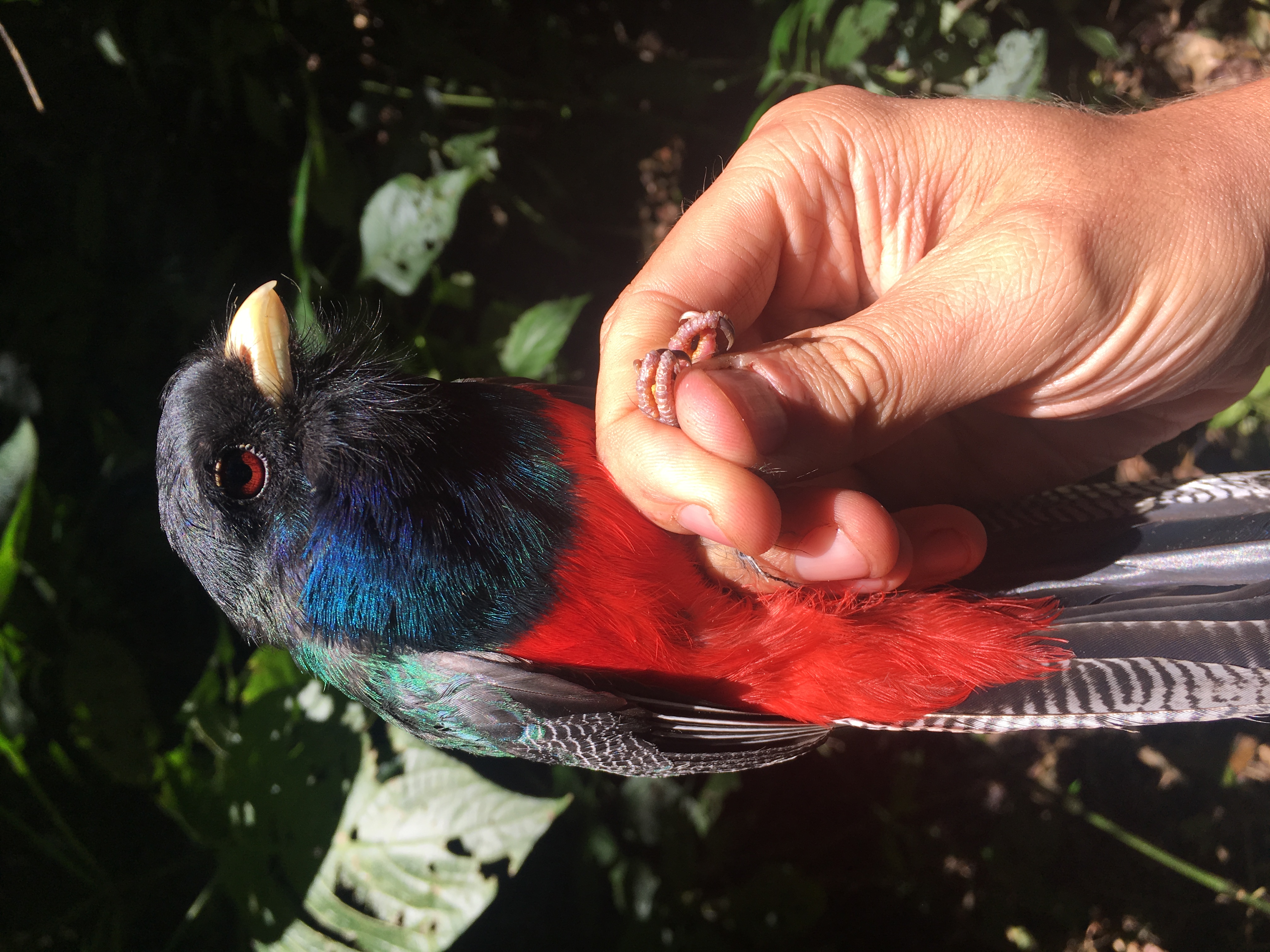

Supplement: S2 Fig — (JPG) [file pone.0248712.s003.JPG]
