# Supplementary material for: Afrotropical montane birds experience upslope shifts and range contractions along a fragmented elevational gradient in response to global warming
Source: PLoS One. 2021 Mar 30;16(3):e0248712. doi: 10.1371/journal.pone.0248712 (PMC8009416; doi:10.1371/journal.pone.0248712)
Supplement: Attachment — Submitted filename: Response to Reviewers.pdf [file pone.0248712.s004.pdf]

Response to Reviewers

Editor:

1. The MS do not have single table and has only two figures. The data can be more appropriately visualized graphically or through tables. Authors can at least provide map of the study area showing current and historical sampling locations (hope they are not exactly the same). I understand that sampling elevations are same but I presume locations must be different. Hence, make this point clear in the methods section and include map as suggested.

We have included in the revised manuscript a map (Fig 2) detailing forest cover in the East and West Usambara Mountains along with the location of the historic and current survey sites. We have also included, in response to Reviewer 3's comments, a conceptual model (Fig 1) comparing predicted impacts of climate change on elevational range shifts of tropical bird species in continuous versus fragmented forest.

2. Since there will be definite LULC change of the landscape between historical period (1979-81) and current (2019), I suggest the authors to prepare LULC map of both time scale and report any change that have undergone during this 40 years period. This will be helpful to understand whether some of the range shifts in birds is due to landuse change rather than climate change per se. Otherwise, how did authors control over land use changes (leading to habitat change) over these years?

We have included in (S1 Fig) time-series aerial imagery for the five forest blocks included in this study. We report in the revised manuscript no change in forest area of the five forest blocks over the study period and minimal change in matrix habitat composition within 500 m of the forest edge. The percent change by area in matrix habitat composition is reported in Table 1.

Reviewer #1

This is a great paper. It clearly describes why the study is important, what the authors found, and what these results mean. I have only one minor comment.

1) Please clarify how elevational shifts were calculated for upper and lower range limits. In particular, it does matter whether all species are included in these calculations or whether species historically found at the lower limit were excluded for lower limit shifts and at upper limit excluded for upper limit shift. This is mentioned in the Discussion but should be incorporated into the more general results as well. Particularly for the upper limits, it makes sense to NOT include species that already live at the highest elevation when calculating upper elevation shifts.

We thank the reviewer for the suggestion. In the revised manuscript, in calculating the upper elevational range limit across species we excluded all species that were recorded at the highest elevation site (2110 m) during both surveys. Similarly, in calculating the lower elevational range limit we excluded all species that were recorded at the lowest elevation site (360 m) during both surveys.

Also please fix the references in text to consistently match the correct style

We have corrected these.

Reviewer #2

My only concern about this manuscript is with the interpretation that these species are moving upslope rather than just disappearing from lower elevations. The study landscape is a very complex one and the West Usambaras (all of the high-elevation sites) and the east Usambaras (all of the low-elevation sites) are separated by a large lowland savannah/agricultural gap and seem very unlikely to have much bird movement between them. Rather, they seem to have independently evolved communities in which all of the movements are likely to have been within them. I therefore wonder if the threat is not much greater than indicated with the east Usambara species simply being pushed upslope with nowhere to go. All of the other comparable paper have dealt with continuous transects in which dispersal is not limited. If you have evidence to the contrary, then I would need to see it to have enough confidence that these bids can in fact cross these gaps. If not, then the analysis needs to be done separately for the two mountain ranges.

Thank you for your comments. In the revised Discussion we highlight that dispersal between forest blocks is highly limited in our study system because of the very limited gap-crossing ability of understory bird species in the Usambara Mountains relative to the distance between forest blocks.

In the revised Results we note that mean upslope elevational range shifts are largely a result of upslope shifts in the lower elevational range of species rather upslope shifts in the upper elevational range of species, and this has resulted in range contractions across species. We further highlight this in a conceptual model that we have presented in the revised Introduction comparing predicted elevational range shifts of understory bird species in continuous versus fragmented forest.

With regard to analysing the EUM and EUM separately, the distance between the 1020 m study site in the EUM and the 1310 m study site in the WUM – 14.2 km – is considerably less than the distance between the 1310 m and 1530 m sites – 24.7 km – both within the WUM, or between the 1530 m site and the 1810/2110 m sites – 16.6 km – both within the WUM, and report this in revised Table 1. To illustrate this further, in the revised text we have added a figure (Fig 2) showing forest cover in the EUM and WUM and the location of survey sites. Finally, we would like to note that matrix habitat composition between all of the forest blocks is the same whether the forest blocks are located in the EUM or WUM or between the EUM and WUM forest blocks: small-scale agriculture in combination with large-scale agriculture (predominantly tea, and sisal at lower elevations), and timber and fuel wood plantations (teak, *Eucalyptus*, pine).

Lastly, analysing the two ranges separately would introduce problems because the upper limits of most species are in the WUM and the lower limits of most species are in the EUM. Thus, separating them would leave little power to test upper range limit shifts in the EUM and lower range limit shifts in the WUM.

Reviewer #3: Summary:

I think this is a solid study that could make a nice contribution to PLoSONE, though with substantive revisions. The dataset is unique and important, the manuscript is well written and the text is balanced. Yet I feel that the manuscript is falling slightly short of the mark, or rather, not expressing its potential, in showing how this helps us understand climate change impacts on tropical species in fragmented

landscapes. This can be improved by first giving more context (in the introduction) for how fragmentation can further disrupt the continuity of populations as they respond to shifting climates (possible predictions and well formulated questions). For example, will fragments experience local environmental changes the same way as large tracts of continuous forest? If not, how are they expected to differ? Rather, most of the introduction text focuses on stating that we don't know much about these systems, which is valid, but there is little development beyond that. Adding more meat here to explore what we do know, about responses to climate change along other montane gradients, and/or responses to fragmentation in other systems, could set up the study better.

We present in the revised Introduction a conceptual model comparing predicted impacts of global warming on elevational range shifts of species in continuous versus fragmented forest. As part of this we highlight in the revised Discussion that the limited dispersal ability of species relative to the distance among forest blocks in the Usambara Mountains is almost certainly an important driver of observed changes in the elevational distribution of understory bird species. We have also added hypotheses to the last paragraph of the Introduction.

Second, I think the authors can do more to dig into the results, e.g., to ask if there are particular characteristics of species that are associated with movements (upslope or downslope, or the extent of shifts). Can we draw generalities on how tropical communities/groups of species/guilds will be impacted? Are there any comparisons that can be made across studies to draw additional insights here? I appreciate that brevity is emphasized, but it seems there is room to address this in more depth in the introduction and discussion, and doing so could make this a stronger contribution.

We have included in the revised Discussion a comparison of species traits among understory bird species that have experienced upslope shifts in mean elevation. We note that there are few species traits that are shared among species in our study system which is also consistent with patterns observed in the Southeast Asia and the Neotropics.

Third, I am left unconvinced regarding the fragmentation component of the study and the role it plays either formally or conceptually in the manuscript. There is not much depth in assessing how fragmentation affects how species shift in their ranges, except to say that the study was performed in a fragmented landscape. The results are considered in the extent of species range shifts compared to those found in other studies, but this is all done without thinking about any specific or general impacts of fragmentation, qualitatively or quantitatively. Would there be a way, for example, to test whether species track expected climate shifts more closely if they are found within the same forest blocks (or conversely, more likely to lag behind expected shifts if they are in different forest blocks)? It seems there could be a way to assess whether species that additionally have to contend with jumping between fragments in order to shift ranges are lagging further behind those that do not (i.e., the species that can move upslope within the same forest block). (It's also possible that I don't fully understand the layout and connectedness of the forest patches in the landscape – I'm just making inferences here based on Table 1).

Thank you for this comment. In the revised manuscript, we have presented a conceptual model predicting the impact of global warming on elevational range shifts of understory bird species in continuous versus fragmented forest.

The elevational ranges of many species in this study typically span 500-1500 m so consequently few shifts have occurred wholly within one forest block. Nonetheless there are a number of shifts that have occurred within one of the largest forest blocks. In the revised Results we report that “Of the six species whose maximum elevation was higher in 2019 than 1980, five were recorded at a higher elevation survey site within the same forest block than they were previously recorded. We recorded only one species, Little Greenbul (*Eurillas virens*), a species that readily crosses forest gaps [21], shifting its maximum elevation upslope over time between forest blocks. By contrast, of the 14 species whose minimum elevation was higher in 2019 than 1980, ten of these 14 upslope shifts were recorded across one or more forest blocks”. Furthermore, we note in the revised Discussion that shift rates in the upper elevational range limit of bird species in Southeast Asia and the Neotropics in continuous forest are higher than that in fragmented forest in the Usambara Mountains.

As an aside, I found the patterns of slower colonization at the upper range limits really interesting. I wonder if there are other behavioral drivers that mediate these movements? Consider conspecific attraction – where individuals are more likely to settle in an area if conspecifics are already present (particularly if the presence of individuals serves as a measure of habitat quality). This could apply to your system, since individuals moving upslope from the lower limit are moving into areas where conspecifics are present, whereas those at the upper range limit are effectively colonizing areas where the species is all together absent or at most at very low densities. If conspecific attraction is important for colonization, this could create a lag in movements at upper limits versus lower ones. Is there any documentation of this elsewhere? It would be an interesting point to entertain briefly in the discussion, space permitting.

We have pointed out in the revised Discussion that colonization of unoccupied higher elevation forest blocks may be inhibited not only by limited dispersal capability of species but also by the absence of attractive conspecific species as well as Allee effects.

Line specific comments and recommendations are below:

Introduction

Lines 51: desvasting -> devastating

Corrected.

Lines 55-56: The authors point out here that we know little about how elevational range shifts may occur in the tropics when species are simultaneously responding to habitat discontinuities and fragmentation. This is an important theme that makes this study unique from others, but there is little other context for how this could compound responses to climate change. What potential impacts have been proposed? What will we learn by doing such studies in fragmented landscapes? I think the overall impact of the study will be higher if this is articulated more, here in the introduction, and connected with some of the ideas put forward in the discussion (or even better if fragmentation can be analyzed formally).

As stated above, we presented in the Introduction a conceptual model (Fig 1) and in the revised Discussion highlight that “habitat fragmentation will almost certainly exacerbate impacts of global warming on shift rates of understory bird species along their lower elevational range limits via lag effects between past habitat loss and species responses [49] as well as edge effects on habitat selection

and nest survival [20,69]. Fragmentation will therefore almost certainly amplify the impacts of global warming on population declines of tropical montane bird species along their trailing edge [15,16]."

Lines 68-74: I encourage the authors to state some hypotheses clearly at the end of the introduction. What are you expecting to see with these surveys? Could you set up the comparison to expected changes due to warming, based on other studies? Having more context ahead of this, as recommended above, would better lead into the questions/aims of the study.

As noted above, we have added a conceptual model with predictions in the revised Introduction and have also incorporated hypotheses in the last paragraph of the revised Introduction.

Line 59-60: Should these references also be numbered?

These are now numbered appropriately.

Methods:

Line 88: What is the "cold season" – is this characterized by less rain? Is this associated with the breeding season for the bird community? Notably, the resurvey is sampling a narrower duration of months compared to the original survey -- are the authors confident that these sampling periods are nevertheless capturing the same season? (In other words, could slight differences in seasonality be an issue here?)

In the revised Methods, we describe temperature differences between the cool and hot season as well as patterns of rainfall in the East and West Usambara Mountains.

The difference in mean daily temperature between the cool and hot seasons in the EUM and WUM recorded over a 22-month period at the 1050 and 1310 m study sites was 3.86 °C. Rainfall in the Usambara Mountains is bimodal with peaks during both the cool and hot season. The breeding season for birds begins the end of August (cool season) and extends through early February (hot season). Although the resurvey effort was conducted over a shorter time period than during the original survey, there are not any distinct seasonality differences that we can identify between the resurvey and the original survey.

Results:

Is there a way of explicitly considering the compositional change or range shifts within and between forest blocks? This seems important to fully base the study on the effects of fragmented forest on movement rates (otherwise the incorporation of fragmentation is a bit superficial, if there is no formal characterization in the study design or data analysis).

As noted above, in the revised Results and Discussion we report the proportion of species whose maximum and minimum recorded elevation shifted between 1980 and 2019, and which occurred within and between forest blocks.

Lines 166-167: Which species were not represented in the resurvey? >13% decline in species richness is substantial. Could additional summaries be given for those species missing from the current survey?

We plan to report these results in greater detail in future publications but can provide the reviewer with a table containing these results if the reviewer would still like to see them.

Discussion:

In the discussion, I would like to see more in-depth evaluation of the kinds of species that move downslope, or those that are more likely to move upslope. Given the possibility for comparisons across three studies (Forero-Medina et al., Freeman & Class, and your study) it seems there is ample opportunity to dig into these patterns a bit more (even while maintaining brevity in the manuscript). I'll reiterate here that this study makes an important contribution to our understanding of upper and lower range limit shifts in tropical systems, particularly by adding a new dataset from the Afrotropics. But the manuscript now stands more as a report on these patterns, yet it could do more to explore the context of patterns, first by setting up clearer questions and possible predictions at the beginning, and then layering some comparisons across studies that considers which species/groups have predictable responses (or not) in your study and across others like this one.

In the revised Discussion we discuss species traits common to species that have shifted their mean elevational range upslope and report there are few common traits. However, because of small sample size (n=29), highlighting differences among species is problematic and note this in the revised Discussion. We are planning, however, to conduct a meta-analysis of species traits based on all studies conducted to date that have examined the impact of climate change on elevational range shifts of bird species in the tropics.

Lines 260-270: Here you report the greater frequency of movements within forest blocks than between forest blocks – so this points to dispersal limitation of species and/or sensitivity to cross gaps. Are these formally reported in the results section? (These kinds of results can put more emphasis on the interactions between fragmentation and climate change in affecting range shifts).

We have moved these findings to the revised Results and the methodology for the calculations to the revised Methods.

Lines 295-298: "Our results suggest that climate warming in combination with average distance among fragments and the limited dispersal ability of many species are very likely important contributory factors to the observed range contractions in bird species in the Usambara Mountains."

Here I don't really think the authors have completely made this case, especially as the results relate to the average distance among fragments. Distance among fragments was not considered explicitly in the analysis itself (I think it should be).

In the revised Discussion we report on the dispersal capability of understory bird species based on (1) long-term (34 years) mark-recapture data of banded birds across a network of 14 forest fragments in the EUM and WUM; (2) radio-tracking of individual gap-adverse species over 8 years; and (3) long-term observations of bird species in matrix habitats. We also report in the revised Discussion on the distance between individual forest blocks relative to the observed dispersal capability of bird species among forest fragments. Finally, in the revised manuscript we include a map of forest cover in the EUM and WUM and the location of survey sites to visually illustrate the distance among the surveyed forest blocks and also include much of this information in revised Table 1.

Lines 320 – 332: I like the emphasis on the high elevation endemics in drawing attention to the conservation priority for this region, though the discussion is necessarily limited since for most of the endemics there are few data available to do a robust analysis. Can the authors address (through personal observation) whether these endemic species are rare numerically, or just not easily detected using the methods employed in this study?

In the revised Discussion we note that of the endemic and endangered species that were historically rare 40 years ago based on both mist-net and line transect surveys conducted by Stuart are also currently so based on mist-net surveys and opportunistic sightings by Newmark and Neate-Clegg.
