# Supplementary material for: Afrotropical montane birds experience upslope shifts and range contractions along a fragmented elevational gradient in response to global warming
Source: PLoS One. 2021 Mar 30;16(3):e0248712. doi: 10.1371/journal.pone.0248712 (PMC8009416; doi:10.1371/journal.pone.0248712)
Supplement: Attachment — Submitted filename: Response to Reviewers.pdf [file pone.0248712.s005.pdf]

Reviewer #1

I like the addition of explicit consideration of elevational range changes in fragmented landscapes. However, and this is a big however, it does mean that the whole pitch of the paper has changed based on the results.

This is an example of hypothesizing after the results are known. The hypothesis here is that fragmentation differentially affects range shifts at lower vs. upper elevation limits via reduced rates of colonization. As is, a reader assumes that the study was designed to test this hypothesis. This is not the case.

Although a conceptual model was not included in our original submission, we believed from the outset that fragmentation would very likely impact elevational range shifts of species. However we did not know to what extent. Thus our conceptual model and hypotheses were not entirely *post hoc*. Furthermore, our inclusion in the revision of the manuscript of this conceptual model and accompanying hypotheses in the Introduction were at the request and recommendation of Reviewer 3.

To fix this, the authors should be open about the relationship between their data and hypotheses about fragmentation. The authors should clearly state (1) what their results were and (2) introduce the hypothesis as a possible explanation of their results [Rather than (1) introduce the hypothesis and (2) describe a test of these predictions]. For example, the authors can say they observed a difference in changes at low vs. high elevation limits towards the end of the Introduction, then subsequently introduce the idea that difficulty of upslope colonizations in fragmented landscapes is a possible explanation for this interesting observation. All text, and the conceptual figure, should stay. Just be clear that these are post-hoc hypotheses. This is perfectly valid, does nothing to detract from the paper, and correctly represents this project [as far as I can judge, based on the original manuscript].

We have incorporated all of these suggested changes in this revision. We have (1) stated in the Introduction that “based on observed elevational range shifts of species in the East and West Usambara Mountains, we predict …”, (2) these predictions have been moved to the last two sentences in the Introduction; and (3) our conceptual model has been renumbered as Figure 2 and follows the last paragraph in the Introduction.

Figure 1 illustrating the fragmented elevation gradient is now presented after paragraph 4 in the Introduction to highlight one of the major themes of this study: the impact of forest fragmentation on the elevational range shifts of tropical montane bird species in response to climate change.

Clarify in Abstract that landscape was already fragmented in the 1970s.

We have stated in the revised Abstract that the elevational gradient has been fragmented over the last 200 years.

Suggestion: Add a figure showing changes in elevations for each species (e.g. each species a column, elevation on the y axis). And/or a strip chart/box plot showing change in total elevational size to illustrate that most species are contracting in range.

Initially we had considered such a figure. However, the main results we report are based on shift rates that have been corrected for differences in sample size between surveys. To produce a plot based on

raw, uncorrected range limits would be somewhat misleading because those values do not account for differences in sample size. We therefore decided instead to plot the corrected shift rates (Figure 3).

Text in Figure 1 low-resolution / hard to read. True for other figures too - make sure final version are high-res.

I believe the pixilation comes from the way that figures are integrated into the reviewer document, having reviewed PLOS ONE articles myself. We will ensure that the final versions are high-resolution.

Beyond the scope of this paper, but interesting to think about specific predictions for range shifts at species' lower limits in fragmented landscapes. Figure 1 illustrates the situation if temperature is the driver (species disappears from lowest fragment and is not present at lowest elevation of second-lowest fragment). One possibility is that upslope shifts at species' lower limits result from being "pushed" up by biotic interactions linked to temperature. In this case, and if dispersal is generally very low (not just for the birds, but also for species whom they interact with in ways important for range limits), then you might expect the species to disappear from the lowest fragment (where it is pushed up) but still live at the lowest elevation of the second-lowest fragment (where there is nothing arriving to "push" it upslope).

We have added in the Introduction and Discussion several sentences discussing the potential effects of competitors and/or natural enemies on range shifts at species' lower limits in fragmented landscapes. However, we find little evidence for this in the Usambara Mountains.
